# The Hydradephaga (Coleoptera, Haliplidae, Gyrinidae, and Dytiscidae) fauna of Cape Breton Island, Nova Scotia, Canada: new records, distributions, and faunal composition

**DOI:** 10.3897/zookeys.897.46344

**Published:** 2019-12-09

**Authors:** Yves Alarie

**Affiliations:** 1 Department of Biology, Laurentian University, Ramsey Lake Road, Sudbury, ON P3E 2C6, Canada Laurentian University Sudbury Canada

**Keywords:** biodiversity, faunistic, Hydradephaga, Maritime Ecozone

## Abstract

The Haliplidae, Gyrinidae, and Dytiscidae (Coleoptera) of Cape Breton Island, Nova Scotia, Canada were surveyed during the years 2006–2007. A total of 2027 individuals from 85 species was collected from 94 different localities, which brings to 87 the number of species recorded for this locality. Among these, *Heterosternuta allegheniana* (Matta & Wolfe), *H. wickhami* (Zaitzev), *Hydroporus appalachius* Sherman, *H. gossei* Larson & Roughley, *H. nigellus* Mannerheim, *H. puberulus* LeConte, *Ilybius picipes* (Kirby), and *I. wasastjernae* (C.R. Sahlberg) are reported for the first time in Nova Scotia. The Nearctic component of the fauna is made up of 71 species (81.6%), the Holarctic component of 16 species (18.4%). Most species are characteristic of both the Boreal and Atlantic Maritime Ecozones and have a transcontinental distribution but 19 species (21.8%), which are generally recognized as species with eastern affinities. In an examination of the Hydradephaga of insular portions of Atlantic Canada, it was shown that the island faunas of Cape Breton Island and Prince Edward Island are very similar (87 and 84 species, respectively) despite differences in composition suggesting that more Hydradephaga species have yet to be found on Cape Breton Island.

## Introduction

Cape Breton Island is a large (10,311 km^2^) rugged and irregularly shaped island, approximately 175 km long by 135 km at is widest, located at 46 degrees latitude, 60 degrees longitude in northern Nova Scotia, Canada at the eastern extremity of the Gulf of St. Lawrence. It lies within the Atlantic Maritime Ecozone along with Québec’s Gaspé Peninsula, Magdalen Islands Archipelago and portions of the south shore of the St. Lawrence River. The climate of this ecozone is strongly influenced by the Atlantic Ocean, which produces cooler summers (average 14 °C) and warmer winters (average -5 °C), with coastal areas having slightly warmer winters and cooler summers than inland. Geologically, this region is a mix of sedimentary and igneous bedrock (Alarie 2016). Cape Breton land mass slopes upward from south to north, culminating in the massive highlands of its northern cape, the highest elevation in the Atlantic region.

Water beetles make up a large part of aquatic invertebrates (Jäch and Balke 2008) and as such they play a vital role in terms of biodiversity and ecosystem functioning, and consequently in the stability of ecosystems (Wallace and Webster 1996). Investigating water beetle assemblages may be particularly illuminating considering the potential these groups are demonstrating as bioindicators of aquatic ecosystem viability (Foster et al. 1990; Fairchild et al. 2000; Lundkvist et al. 2001; Arnott et al. 2006). They also allow the diagnosis of alterations causes, the establishment of criteria for protection and restoration of interesting ecosystems and finally the integrated management of watersheds (Abellán et al. 2007). Thus, good knowledge of the species presence and distribution is necessary to protect biodiversity (Millán et al. 2014). Moreover, primary biodiversity data represent the fundamental elements of any study in systematics and evolutionary processes (May 1990; Funk and Richardson 2002; Hortal et al. 2015).

Investigations of the Hydradephaga (Dytiscidae, Haliplidae, Gyrinidae) of the Canadian Maritimes have been sporadic and regionally variable. Recent papers (Majka 2008; Majka and Kenner 2009; Alarie 2009, 2016; Majka et al. 2009; Webster et al. 2016) resulted in a better understanding of the Hydradephaga fauna in some areas. Despite this rapid increase in knowledge of faunal composition, there are still many Hydradephaga species waiting for discovery in eastern Canada. This is amply evidenced by the fact that 30 species were recently added to the list of Prince Edward Island (Alarie 2016).

Little is known about the Hydradephaga fauna of Cape Breton Island. Prior to this study, 51 species were reported as valid records in the faunal list for Cape Breton Island (Alarie 2016), a small proportion of the 118 (43.2%) reported in Nova Scotia (Bousquet et al. 2013). This study was conducted as part of a comprehensive baseline field survey of Hydradephaga biodiversity of Canadian Maritimes Islands (Alarie 2009, 2016). Its main objective is to improve knowledge of the Hydradephaga of Cape Breton Island. Of particular interest was the identification of new species additions to the known fauna of Nova Scotia.

## Materials and methods

### Study areas

All of Nova Scotia mainland and Cape Breton Island sit within the Acadian Forest region as described by Rowe (1972), which has a mixed-forest species composition consisting predominately of conifers, especially on sites where drainage is impeded. The major conifers include red, white, and black spruce; balsam fir; eastern white and red pine; and eastern hemlock. Common hardwoods include red and sugar maple; white and yellow birch; trembling and largetooth aspen; and beech (Neily et al. 2005).

Although physically separated from the Nova Scotia peninsula by the Strait of Canso, Cape Breton is artificially connected to mainland Nova Scotia by the Canso Causeway. The island is located east-northeast of the mainland with its northern and western coasts fronting on the Gulf of Saint Lawrence; its western coast also forming the eastern limits of the Northumberland Strait. The eastern and southern coasts front the Atlantic Ocean; its eastern coast also forming the western limits of the Cabot Strait. Cape Breton Island is composed mainly of rocky shores, rolling farmland, glacial valleys, barren headlands, mountains, woods and plateaus. The boreal highlands of Cape Breton reach elevations of 300–500 m and represent true boreal forest habitat, which is rare in Nova Scotia (Neily et al. 2005). Geological evidence suggests that at least part of Cape Breton was originally joined with present-day Scotland and Norway (www.newworldencyclopedia.org/entry/Cape_Breton_Island).

Cape Breton Island’s hydrological features include the Bras d’Or Lake system, a salt-water fjord at the heart of the island, and freshwater features including Lake Ainslie, the Margaree River system, and the Mira River. Innumerable smaller rivers and streams drain into the Bras d’Or Lake estuary and onto the Gulf of St. Lawrence and Atlantic coasts (www.newworldencyclopedia.org/entry/Cape_Breton_Island).

Geographically, Cape Breton Island is subdivided into four counties: Cape Breton, Inverness, Richmond, and Victoria. More than 70% of the total Cape Breton population live in the industrialized Cape Breton County. The boreal highlands of Cape Breton are located in the northern parts of Victoria and Cape Breton Counties. The climate of this region is influenced by the higher elevations, strong ocean winds and heavy blankets of dense fog that occur during spring and summer. The headwater streams of the highland regions flow over a primarily ancient metamorphic and granitic dominated geologic landscape, originating from cool springs or draining acidic, sphagnum bogs (Ogden et al. 2018). Inverness and Richmond Counties are largely rural and boast Nova Scotia’s most pristine areas. Located within Inverness County, the Margaree River is one of the world’s most famous fishing rivers. There are almost no lakes in this region, but there are many steep-fast flowing rivers and streams. Inverness County includes some of the most interesting old forests and undisturbed areas in Nova Scotia. Richmond County is the least well-known county in all Nova Scotia in terms of its beetle fauna. There are many lakes, marshes, and bogs in this area, which have been very little investigated.

### Collecting methods

Collections were conducted over three periods, 05–10 May 2006, 17–22 August 2006, and 14–19 May 2007. Sampling was unstructured and qualitative with the goal of obtaining a strict inventory of Hydradephaga of Cape Breton Island. Beetles were collected using D-net sweeps in a variety of microhabitats including macrophyte beds, rocky shores, organic-rich sediments, and open water. Overall 94 samples were obtained, which are listed in Table 1, along with locality data and habitat information.

**Table 1.** Cape Breton Island, Nova Scotia (Canada) sampling localities and habitats (2006–2007): letter in sample code refers to the county. Key: C = Cape Breton; I = Inverness; R = Richmond; V = Victoria.

Sample	Locality	Habitat
C01	Cape Breton Co., Leitches creek Road, 1 km off Hwy 223. 14.v.2007	Bog lake with *Carex* and Ericaceae along margin
C02	Cape Breton Co., Quarry Road, off Leitches creek Road, ca. 6 km off Hwy 223. 14.v.2007	Shallow creek in spruce forest; bed with big boulders; swift current; littoral zone with alder, beech, yellow birch
V03	Victoria Co., Mackillop Road off Hwy 105 at exit to Cabot trail. 15.v.2007	Bog pool on *Sphagnum* bed in white spruce forest; dark water; *Carex* along littoral zone
V04	Victoria Co., St. Ann’s Provincial Park, Mackillop Road off Hwy 105. 15.v.2007	Pothole along shore of stream; mats of Graminea and *Scirpus*
V05	Victoria Co., Meadow Road, 4 km off Cabot trail N. 15.v.2007	Shallow creek with swift current, ca. 3–4 m wide; cold water
V06	Victoria Co., Meadow Road, 5 km off Cabot trail N. 15.v.2007	Shallow creek
V07	Victoria Co., Oregon Road, 2 km off Cabot trail N. 15.v.2007	Pond covered with dead *Scirpus*
V08	Victoria Co., Cabot trail N., near junction to Meadow Road. 16.v.2007	Shallow creek on rocky bed, with *Sphagnum* and bryophytes; spruce forest with *Fagus*; cold water (6 C)
V09	Victoria Co., West Tarbot Road ca. 1 km off Cabot trail N. 16.v.2007	Ephemeral pot holes on clay bed, in *Scirpus* and Graminea field; shallow with algae; white spruce forest
V10	Victoria Co., West Tarbot Road ca. 1km off Cabot trail N. 16.v.2007	Ephemeral roadside ditch; very eutrophic, with heavy accumulation of *Sphagnum*, black sediments; dark water
V11	Victoria Co., West Tarbot Road ca. 6 km off Cabot trail N. 16.v.2007	Roadside ditch, slowly moving water; very shallow potholes (ca. 6 cm) with emerging vegetation
V12	Victoria Co., Cabot trail N., near junction Tarbot Vale Road and Rear Barachois Road. 16.v.2007	Large pools formed by the river; clear water; rocky bed covered with organic matters (dead wood, dead leaves) in white spruce forest
V13	Victoria Co., Cabot trail N., 4 km south of Little River. 16.v.2007	Man-made pond with mats of *Scirpus*
V14	Victoria Co., Cabot trail N., 4 km S. Little River. 16.v.2007	Fen with Graminea
V15	Victoria Co., Rear Little River Road, off Cabot trail N. 16.v.2007	Brook flowing over rocky bed, ca. 2 m wide; moderately moving water
C16	Cape Breton Co., Morrisson Road, ca. 6 km off Hwy 22 S. 18.v.2007	*Sphagnum* bog in white spruce forest; with Ericacea, *Scirpus* and *Typha*
C17	Cape Breton Co., Morrisson Road, ca. 7 km off Hwy 22 S. 18.v.2007	*Sphagnum* pool with Ericacea and *Scirpus* in white spruce forest
C18	Cape Breton Co., Morrisson Road, ca. 8 km off Hwy 22 S. 18.v.2007	Shallow roadside ditch with Graminea and *Sphagnum*
C19	Cape Breton Co., Broughton Road near junction to Morrisson Rd. 18.v.2007	Shallow eutrophic brook flowing over rocky bed
C20	Cape Breton Co., Broughton Road, 2 km off junction to Morrisson Rd. 18.v.2007	Eutrophic lake
C21	Cape Breton Co., Broughton Road, 3 km off junction to Morrisson Rd. 18.v.2007	*Sphagnum* bog
C22	Cape Breton Co., Broughton Road, 4 km off junction to Morrisson Rd. 18.v.2007	Road-side ditch
C23	Cape Breton Co., South Head, Sailor Dans Lane. 18.v.2007	Brook with heavy accumulation of bryophytes
C24	Cape Breton Co., South Head, Sailor Dans Lane. 18.v.2007	Cattail pond with bryophytes and *Carex*
C25	Victoria Co., West Side Baddek Road, junction Hunter Mtn Road. 19.v.2007	Small eutrophic creek; heavy accumulation of organic debris; margin with dense vegetation including dead Graminea
V26	Victoria Co., West Side Baddek Road, junction Hunter Mtn Rd. 19.v.2007	Fen with dark brown water; heavy accumulation of Graminea
V27	Victoria Co., Baddek Forks. 19.v.2007	Ephemeral woodland pool with *Scirpus*; bed with heavy accumulation of dead leaves
V28	Victoria Co., Baddeck River at Baddek Forks. 19.v.2007	Pools beside river; very eutrophic
R29	Richmond Co., Road off Hwy 104^E^ at exit 44 to Port Malcom. 05.v.2006	Roadside bog with *Sphagnum* and *Typha*; in *Picea* and *Larix laricina* forest
R30	Richmond Co., Road off Hwy 104^E^ near Port Hawskberry. 05.v.2006	Roadside ditch on rocky bed covered with mud; shore with *Typha* and *Alnus*
R31	Richmond Co., Road off Hwy 104^E^ towards Isle Madame. 05.v.2006	Shallow creek on rocky bed, with mats of algae; shoreline with *Carex* and Graminea
R32	Richmond Co., Road off Hwy 104^E^ towards Isle Madame. 05.v.2006	Large creek flowing over rocky bed, presence of algae; in *Picea* and *Betula allegheniensis* forest; shoreline covered with dense bryophytes
R33	Richmond Co., Road off Hwy 104^E^ towards Isle Madame. 05.v.2006	Pond with heavy accumulation of organic debris
R34	Richmond Co., Isle Madame, Lake road off Hwy 206. 06.v.2006	Man-made pond; rocky bed
R35	Richmond Co., Isle Madame, Lake road off Hwy 206. 06.v.2006	Lake on sandy bed
R36	Richmond Co., Isle Madame, Hwy 206 at Anthony road. 06.v.2006	Shallow puddle on muddy bed, in Graminea field
R37	Richmond Co., Isle Madame, Hwy 320 West at bridge, ca. 4 km East of D’Escousses. 06.v.2006	Eutrophic creek
R38	Richmond Co., Isle Madame, Hwy 320 West, ca. 1 km West of D’Escousses. 06.v.2006	Pool with dark brown water, in a vast field of *Carex*; *Larix laricina* present
R39	Richmond Co., Isle Madame, Hwy 320 West, ca. 2 km West of D’Escousses. 06.v.2006	Shallow cattail pond in *Picea* forest
R40	Richmond Co., Sporting Mountain Road, ca. 2 km off Hwy 4 at exit 47. 06.v.2006	*Sphagnum* bog in *Picea* forest.
R41	Richmond Co., Sporting Mountain Road, ca. 3 km off Hwy 4 at exit 47. 06.v.2006	Small creek flowing on rocky bed with dense mats of *Sphagnum*/bryophytes; in *Abies balsamifera* and *Betula allegheniensis* forest
R42	Richmond Co., Sporting Mountain Road, dead end of Hwy 4 at exit 47. 06.v.2006	Man-made shallow pond
R43	Richmond Co., Road off Sporting Mountain Road, towards St Peters lake. 06.v.2006	Lake; shoreline with *Sphagnum*
R44	Richmond Co., Fleur-de-Lis trail, 3 km East of Grand River. 07.v.2006	Road-side bog ditch with slow-moving dark brown water; heavy accumulation of *Sphagnum* and *Scirpus*.
R45	Richmond Co., Fleur-de-Lis trail at junction Barren Hill Road, ca. 6 km East Grand River. 07.v.2006	*Carex* pool with *Saricena purpurea*
R46	Richmond Co., Fleur-de-Lis trail, ca. 9 km E. Grand River. 07.v.2006	Pond in *Picea* forest; littoral margin with abundance of *Scirpus* and Ericacea
R47	Richmond Co., Fleur-de-Lis trail, ca. 6 km East of St. Esprit. 07.v.2006	Inundated *Picea* forest; dark brown slow-moving water
R48	Richmond Co., North Framboise, 5 km W. off Fleur-de-Lis trail. 07.v.2006	Small roadside ditch with Graminea
C49	Cape Breton Co., East Bay, Morrison Road, off Hwy 4. 08.v.2006	Shallow pond fed with flowing water; rocky bed; clear water; dense Graminea along shoreline
C50	Cape Breton Co., East Bay, Morrison Road, off Hwy 4, past bridge. 08.v.2006	Ephemeral pond with accumulation of dead leaves, in *Acer*, *Fagus*, and *Abies* forest
C51	Cape Breton Co., East Bay, Morrison Road, off Hwy 4, at bridge. 08.v.2006	Discharge of lake; pond-like, very eutrophic; dense Graminea along shoreline
C52	Cape Breton Co., East Bay, Morrison Road, off Hwy 4, at bridge. 08.v.2006	Shallow creek flowing over rocky bed; about 1 m wide
C53	Cape Breton Co., East Bay, Chapei Road, off Meadows Road, about 7 km S. of Hwy 4. 08.v.2006	Ephemeral very humic pond with dead leaves; very dark water; shoreline with dense mats of bryophytes
C54	Cape Breton Co., Rear Big Pond Road, 6 km off junction with Chapei Road. 08.v.2006	Small creek flowing over rocky bed
C55	Cape Breton Co., Rear Big Pond Road, 5 km off junction with Chapei Road at Big Pond. 08.v.2006	Muddy creek with cold water; shoreline with Graminea and *Sphagnum*; in *Picea* and *Abies balsamea* forest
C56	Cape Breton Co., Rear Big Pond Road, 1 km off junction with Chapei Road. 08.v.2006	Man-made pond; full of organic debris
C57	Cape Breton Co., Frank Macdonald Road, 9 km off junction with Soldier Cave Road, off Hwy 4. 09.v.2006	*Sphagnum* bog
C58	Cape Breton Co., Frank Macdonald Road, 9 km off junction with Soldier Cave Road, off Hwy 4. 09.v.2006	Roadside *Typha* pond
C59	Cape Breton Co., Frank Macdonald Road, 8 km off junction with Soldier Cave Road, off Hwy 4. 09.v.2006	*Typha* pond
C60	Cape Breton Co., Frank Macdonald Road, 5 km off junction with Soldier Cave Road, off Hwy 4. 09.v.2006	Shallow narrow creek with bryophytes.
C61	Cape Breton Co., Frank Macdonald Road, 4 km off junction with Soldier Cave Road, off Hwy 4. 09.v.2006	Shallow *Carex* puddles
R62	Richmond Co., Loch Lamond West Road, 11 km of Grand River. 09.v.2006	Emissary of Loch Lamond lake; collecting along river arms; shoreline covered with vegetation
R63	Richmond Co., Loch Lamond Road, 14 km of Grand River. 09.v.2006	Pools covered with bryophytes; dark brown and cold water
R64	Richmond Co., Loch Lamond Road, 14 km of Grand River. 09.v.2006	Small creek flowing over rocky bed
R65	Richmond Co., Loch Lamond Road, 16 km of Grand River. 09.v.2006	Fen; dense accumulation of Graminea and bryophytes; very dark water
I66	Inverness Co., Greignish Mtns Road, 1 Km off junction Hwy 4B. 10.v.2006	Pond
I67	Inverness Co., Road 104E, 2 km off junction to Greignish Mtns Road, off Hwy 19 at Greignish. 10.v.2006	Fen with *Scirpus* and mats of bryophytes; dark brown water
I68	Inverness Co., Road 104E, 5 km off junction to Greignish Mtns Road, off Hwy 19 at Greignish. 10.v.2006	Small brook in *Sphagnum* bog
I69	Inverness Co., Road 104E, 12 km off junction to Greignish Mtns Road, off Hwy 19 at Greignish. 10.v.2006	Shallow pools with mats of Graminea and heavy accumulation of dead maple leaves; higher elevation
I70	Inverness Co., Road 104E, 4 km off junction to Greignish Mtns Road, off Hwy 19 at Greignish. 10.v.2006	Bog with *Carex* and *Saracenia purpurea*
I71	Inverness Co., Road 104E, off junction to Greignish Mtns Road, off Hwy 19 at Greignish. 10.v.2006	Bog with *Carex*
I72	Inverness Co., Graham River, at J. D. MacDonald Road near Judique South Hwy 19. 10.v.2006	Stream flowing over rocky bed
I73	Inverness Co., Margaree River North East, off Cabot Trail. 17.viii.2006	Larger river flowing over rocky bed
I74	Inverness Co., Ingram Charlie Brook, East Big Interval Road at bridge, 7 km of East Margaree Valley. 17.viii.2006	Brook flowing over big boulders covered with bryophytes
I75	Inverness Co., East Big Interval Road, 12 km of East Margaree Valley. 17.viii.2006	Small brook; beetles collected underneath the banks
I76	Inverness Co., East Big Interval Road, ca. 12 km of East Margaree Valley. 18.viii.2006	Beaver dam ditch; dense mats of *Carex* along shoreline
I77	Inverness Co., East Big Interval Road, ca. 21 km of East Margaree Valley. 18.viii.2006	Small creek with very slow-moving water (almost still); muddy bed, dark brown to black sediment
I78	Inverness Co., Kingross Crossing Road, ca. 1 km off East Big Interval Road, ca. 20 km of East Margaree Valley. 18.viii.2006	Small pool with crystal clear water; pool likely formed from a brook
I79	Inverness Co., East Big Interval Road, ca. 2 km off Kingross Crossing Road. 18.viii.2006	Small beaver dam pool, almost still water, fed from a small creek; muddy bottom, dark brown to black sediment
I80	Inverness Co., North of St. Joseph du Moine, Bazile Road, off Cabot trail at bridge. 21.viii.2006	Small eutrophic creek; flowing water at the middle over rocky bed; shoreline with dense vegetation (*Eupatorium maculatum*; *Equisetum* sp.; muddy shoreline; beetles collected along shoreline in shallow water
I81	Inverness Co., North of Saint Joseph du Moine, Bazile Road, ca. 8 km off Cabot trail. 21.viii.2006	Small creek with big boulders; swift currents
I82	Inverness Co., North of Saint Joseph du Moine, Bazile Road, off Cabot trail. 21.viii.2006	Lake; littoral zone with *Typha*; muddy
I83	Inverness Co., North of Gold Brook, Cabot trail. 21.viii.2006	Roadside ditch; shoreline with *Spirea*, *Alnus*, and *Carex*; heavy accumulation of dead leaves
V84	Victoria Co., Middle River, at Cabot trail. 21.viii.2006	Roadside ditch with slow moving water; shoreline with *Alnus* and *Carex*
V85	Victoria Co., Egypt Road, at Cabot trail. 21.viii.2006	Eutrophic brook with swift current; rocky bed; presence of algae
I86	Inverness Co., Cranton Cross Road, off Margaree Centre. 21.viii.2006	Brook with crystal clear water; almost still water
I87	Inverness Co., Southwest Margaree Road, ca. 4 km south of Cabot trail. 22.viii.2006	Roadside ditch in spruce forest
I88	Inverness Co., Southwest Margaree Road, ca. 8 km south of Cabot trail. 22.viii.2006	Small eutrophic creek with dense vegetation along shoreline; deep, with slow moving water; rocky bed covered with sediments
I89	Inverness Co., Hwy 395 off Hwy 19. 22.viii.2006	Small creek with swift current; cold water, large boulders; abundance of *Mentha* along shoreline
I90	Inverness Co., south west Margaree River at Bridge, Hwy 395 at junction to Kiltarlity Road. 22.viii.2006	Arm of the river looking like a large ditch; shallow with clear water; dense vegetation (*Carex*, *Scirpus*, *Myositis*) along shoreline; accumulation of algae in the middle; extremely beetle rich
I91	Inverness Co., south west Margaree River at Bridge, Hwy 395 at junction to Kiltarlity Road. 22.viii.2006	I have sampled into algae along shoreline of the river
I92	Inverness Co., Kiltarlity Road. 22.viii.2006	Lake with clear water; *Typha* and nenuphar along shoreline
I93	Inverness Co., Scotsville, junction Hwy 395 and Scotsville Road at bridge. 22.viii.2006	Emissary of lake Ainslie
I94	Inverness Co., near junction to Mountain Road and Scotsville Road. 22.viii.2006	Shallow pond in open prairie overlooking lake Ainslie; main vegetation: *Equisetum*, *Typha* and *Carex*

### Nomenclature

Nomenclature is based on the classification in Oygur and Wolfe (1991) (Gyrinidae: *Gyrinus* Müller), Vondel (2005) (Haliplidae), Nilsson and Hájek (2019) (Dytiscidae) and Gustafson and Miller (2015) (Gyrinidae: *Dineutus* MacLeay).

### Depositories

Voucher specimens are deposited in the author’s research collection (Department of Biology, Laurentian University, Sudbury, Ontario).

## Results

In total, 2027 specimens representing 85 species of Hydradephaga were collected in this study (Table 2). Among these, eleven species are reported for the first time for Nova Scotia. Details of species added to the Nova Scotia’s fauna follow.

**Table 2.** Species of Hydradephaga (Dytiscidae, Gyrinidae, Haliplidae) collected in Cape Breton Island, Nova Scotia, Canada in 2006 and 2007 with sample numbers (as in Table 1), absolute (AF) and relative frequencies (%), and relative frequency of occurrence (RFO). Species in **bold** denote new records from Nova Scotia given in the present account.

Taxon	Sample numbers	AF (%)	RFO
** Haliplidae **			
*Haliplus canadensis* Wallis	C25, I78, I88	5 (0.25)	3.19
*Haliplus connexus* Matheson	I79, I80, I93	4 (0.20)	3.19
*Haliplus cribarius* LeConte	R62, I79	3 (0.15)	2.13
*Haliplus fulvus* (Fabricius)	R45, C42, I78, I79, I90	11 (0.54)	5.32
*Haliplus immaculicollis* Harris	V05, V11, V13, C25, V28, R37, R42, R44, C49, C58, R62, I69, I76, I77, I78, I79, I80, V84, I86, I87, I88, I90, I91, I92, I93	112 (5.53)	26.60
*Haliplus longulus* LeConte	R45, I71	2 (0.10)	2.13
*Peltodytes edentulus* (LeConte)	I91, I93	28 (1.38)	2.13
** Dytiscidae **			
*Acilius mediatus* (Say)	V09, I83, I87	5 (0.25)	3.19
*Acilius semisulcatus* Aubé	V04, R34, R39, I67, I92	7 (0.35)	5.32
*Agabus ambiguuus* (Say)	V04, V09, V10, C25, V26, V28, C20, C49, I67, I76, I79, I80, I82, I83, V84, I90	76 (3.75)	17.02
*Agabus anthracinus* Mannerheim	V03, C21, C22, C24, V26, V28, R29, R40, R46, R47, C49, C57, C59, C61, R63, R65, I67, I69, I76, I80	95 (4.69)	21.28
*Agabus erythropterus* (Say)	V09, C25, R46, R48, I76, I77, I78, I79, I86; I90	76 (3.75)	10.64
*Agabus leptapsis* (LeConte)	V12, I75, I76, I79, I80	5 (0.25)	5.32
*Agabus phaeopterus* (Kirby)	V27	1 (0.05)	1.06
*Agabus semipunctatus* (Kirby)	V03, C16, C24, V27, R46, C59	6 (0.30)	6.38
*Agabus subfuscatus* Sharp	R29, R44, C61, R65, I66	13 (0.64)	5.32
*Clemnius laccophilinus* (LeConte)	I92	1 (0.05)	1.06
*Colymbetes paykulli* Erichson	V03, C61	2 (0.10)	2.13
*Colymbetes sculptilis* Harris	I76, I80	2 (0.10)	2.13
*Copelatus glyphicus* (Say)	V03, V04, V07, V27, C17, C18, C19, C24, R31, R44	64 (3.16)	10.64
*Coptotomus longulus* LeConte	C21, R33	2 (0.10)	2.13
*Desmopachria convexa* (Aubé)	C21, C24, R40, R45, R46, C57, C58, C59, I69, I70, I71, I94	70 (3.45)	12.77
*Dytiscus fasciventris* Say	V84, I88, I92	4 (0.20)	3.19
*Dytiscus verticalis* Say	R46, I69	2 (0.10)	2.13
***Heterosternuta allegheniana* (Matta & Wolfe)**	**R31, R32, R64, I72**	**39 (1.92)**	**4.26**
*Heterosternuta pulchra* (LeConte)	R32, I73, V85, I88, I89	28 (1.38)	5.32
***Heterosternuta wickhami* (Zaitzev)**	**I78, I79, V85**	**9 (0.44)**	**3.19**
*Hydaticus aruspex* Clark	C24, V26, C51, R65, I67, I70, I71, I94	9 (0.44)	8.51
*Hydrocolus paugus* (Fall)	V03, C17, V27, R40, I67, I75, I80, V84	12 (0.59)	8.51
*Hydrocolus stagnalis* (G. & H.)	V08, C18, C19, R46, C50, I90	7 (0.35)	6.38
***Hydroporus appalachius* Sherman**	**I79, I86**	**33 (1.63)**	**2.13**
*Hydroporus badiellus* Fall	R40, C57, I70	13 (0.64)	3.19
*Hydroporus dentellus* Fall	V09, V28, R40, R45, R46, R47, R65, I92, I93	24 (1.18)	9.58
***Hydroporus gossei* Larson & Roughley**	**V12, V26, V27, C53, C59, I88, I90**	**11 (0.54)**	**7.45**
***Hydroporus nigellus* Mannerheim**	**C21, V28**	**2 (0.10)**	**2.13**
*Hydroporus niger* Say	C49, C59, I67, I88	7 (0.35)	4.26
*Hydroporus notabilis* LeConte	V04, C18, C21, C24, V26, V27, V28, R42, C49, C61, I69, I76, I78, I80, I81, I83, V84, I87, I88, I90, I93, I94	52 (2.57)	23.40
*Hydroporus obscurus* Sturm	I70	1 (0.05)	1.06
***Hydroporus puberulus* LeConte**	**C53, I71**	**9 (0.44)**	**2.13**
*Hydroporus rufinasus* Mannerheim	R29, R45, R65	4 (0.20)	3.19
*Hydroporus signatus* Mannerheim	V07, V10, C18, C17, C21, C24, V28, R29, R42, R46, R47, C49, C59, C61	34 (1.68)	14.89
*Hydroporus striola* (Gyllenhal)	V03, V07, V26, V28, C20, R30, R32, R36, R38, R45, R46, C49, C53, C59, C61, I80, I83, V84, I88, I90	56 (2.76)	21.28
*Hydroporus tenebrosus* LeConte	C18, C53, I93	4 (0.20)	3.19
*Hydroporus tristis* (Paykull)	V07, V09, V14, C17, C18, C21, C24, V27, C50, C53, C59, C61, I77, I80, I83	44 (2.17)	15.96
*Hygrotus impressopunctatus* (Schaller)	R35	1 (0.05)	1.06
*Hygrotus picatus* (Kirby)	V04, C20, R46, R47, I76, I94	8 (0.40)	6.38
*Hygrotus sayi* Balfour-Browne	V14, C25, V26, V28, R45, R47, C58, I80, I87, I88, I94	25 (1.23)	11.70
*Hygrotus turbidus* (LeConte)	R39; I66	11 (0.54)	2.13
*Ilybiosoma seriatum* (Say)	C02, V05, V08, C19, C23, C25, R31, C50, C52, C54, C60, R64, I67, I68, I74, I75, V85	76 (3.75)	18.09
*Ilybius angustior* (Gyllenhal)	R39, R46, I83,	6 (0.30)	3.19
*Ilybius biguttulus* (Germar)	V03, V04, C21, C24, V28, R34, C49, C59, R64, I75, I76, I77, I80, I87, I88, I90, I92, I93, I94	106 (5.23)	20.21
*Ilybius confusus* Aubé	I80	1 (0.05)	1.06
*Ilybius discedens* Sharp	V27, R29, R46, C49, C57, I71, I92	11 (0.54)	7.45
*Ilybius erichsoni* G. & H.	V07, C53	7 (0.35)	2.13
*Ilybius ignarus* (LeConte)	V03, C21, V27 R45	6 (0.30)	4.26
*Ilybius larsoni* (Fery & Nilsson)	V04, V06, V09, C18, C21, V27, R40, R48, C50, C61, I74	22 (1.09)	11.7
***Ilybius picipes* (Kirby)**	**V26, I76**	**6 (0.30)**	**2.13**
*Ilybius pleuriticus* (LeConte)	C56, C59, I76, I87, I92	15 (0.74)	5.32
***Ilybius wasastjernae* (C.R. Sahlberg)**	**V27**	**1 (0.05)**	**1.06**
*Laccophilus m. maculosus* Say	V13, C20, C22, C24, V28, R33, R34, R35, R42, C49, I66, I69, I80, I87	32 (1.58)	14.89
*Laccornis latens* (Fall)	R29	6 (0.29)	1.06
*Liodessus affinis* (Say)	V04, V26, V28, R34, R35, R42, C49, C59, R62, I90, I93	55 (2.71)	11.70
*Meridiorhantus sinuatus* (LeConte)	R38, C52	3 (0.15)	2.13
*Nebrioporus rotundatus* (LeConte)	C25, R32, I73, I88	35 (1.73)	4.26
*Neoporus carolinus* (Fall)	V09, V12, V13, V15, V28, R40, R41, C49, C50, C52, C55, C57, I68, I75, I76, I77, I78, I79, I80, I86, I88	97 (4.79)	22.34
*Neoporus clypealis* (Sharp)	R32, R62, I80, I88, I93	14 (0.69)	5.32
*Neoporus dimidiatus* (G. & H.)	V28, I77, I78, I79, V84, I86, I88, I90, I91	44 (2.17)	9.58
*Neoporus spurius* (LeConte)	I80, V85, I91	15 (0.74)	3.19
*Neoporus sulcipennis* (Fall)	R64, 172, I80, I88, I89	28 (1.38)	5.32
*Neoporus undulatus* (Fall)	C25, V28, R35, R43, R45, R47, I80, I87, I90, I92, I93	84 (4.14)	11.70
*Oreodytess. scitulus* (LeConte)	I72, I73	8 (0.40)	2.13
*Platambus obtusatus* (Say)	V26, R38, R40, C50, C61, R64, I83	9 (0.44)	7.45
*Rhantus binotatus* (Harris)	V04, V07, V26, R34, I67, I80, I83, V84, I94	15 (0.74)	9.58
*Rhantus suturellus* (Harris)	V28, R46, R65	4 (0.20)	3.19
*Rhantus wallisi* (Harris)	C24, R45, C59, C56, I66	11 (0.54)	5.32
** Gyrinidae **			
*Dineutus hornii* Roberts	CO1, R35, I93	17 (0.84)	3.19
*Dineutus nigrior* Roberts	R34, R35, C56	6 (0.30)	3.19
*Gyrinus affinis* Aubé	R40, C53, C56, I76,	32 (1.58)	4.26
*Gyrinus aquiris* LeConte	C01, R45, R46; I90	9 (0.44)	4.26
*Gyrinus confinis* Fall	R45	1 (0.05)	1.06
*Gyrinus fraternus* Couper	R45; R62	16 (0.79)	2.13
*Gyrinus gehringi* Chamberlain	V07, V13, R46, C53, I66, I76, V84, V85	48 (2.37)	8.51
*Gyrinus latilimbus* Fall	V13, R45, C56, R62, I66, I76, I79	44 (2.17)	7.45
*Gyrinus pugionis* Fall	C01, V14, R45, I92, I93	69 (3.40)	5.32
*Gyrinus sayi* Aubé	V13, C21, R34, R35, R40, R45, R46, R47, C53, R62, I67, I76	29 (1.43)	12.77
	Total	2027	

### Heterosternuta allegheniana

Taxon classificationAnimaliaColeopteraDytiscidae

(Matta & Wolfe)

B242F0C1-055B-5965-84E9-299B05BEC324

#### Notes.

This species is reported from 39 specimens collected in Richmond County and Inverness County (samples R31, R32, R64, I72).

#### Habitat.

All specimens were collected on pebble substrate or in leaf litter along the margin of cold creek and streams in accordance with Matta and Wolfe (1981).

#### Distribution in the Maritime Ecozone.

Prior to this study, *Heterosternuta allegheniana* had only been reported from New Brunswick and southern Québec (Bousquet et al. 2013). Its presence in Nova Scotia therefore represents its easternmost distribution in Canada.

### Heterosternuta wickhami

Taxon classificationAnimaliaColeopteraDytiscidae

(Zaitzev)

F9BFC362-3739-5770-810E-62CC0C180D6E

#### Notes.

This species is reported from nine specimens collected in Victoria County and Inverness County (samples I78, I79, V85).

#### Habitat.

Like the previous species all specimens were collected in gravel along the margins of streams. Matta and Wolfe (1981) state this species is most common at the margin of medium to small streams.

#### Distribution in the Maritime Ecozone.

This is the first record of *H. wickhami* in the Maritimes. Prior to this study it had only been reported from Ontario and Québec. Its presence on Cape Breton Island represents a significant extension of this species to eastern Canada (Bousquet et al. 2013).

### Hydroporus appalachius

Taxon classificationAnimaliaColeopteraDytiscidae

Sherman

BB488C4B-261D-5C54-B450-0DD522BCB829

#### Notes.

Several specimens of this distinctive species were collected at two different sites in Inverness County (samples I79, I86).

#### Habitat.

*Hydroporus appalachius* is usually found in habitats where there are some water movements either along the margins of small lakes or in small streams and springs (Larson et al. 2000), which describe exactly the habitats where these beetles were found in Cape Breton Island.

#### Distribution in the Maritime Ecozone.

This species has a wide range in North America east of the Rocky Mountains. It occurs from Labrador and New Hampshire west to the northern Great Plains and north into the boreal zone and southern limits of the low artic (Larson et al. 2000). Its presence in Cape Breton Island represents the first mention of the species in the Canadian Maritimes (Bousquet et al. 2013).

### Hydroporus gossei

Taxon classificationAnimaliaColeopteraDytiscidae

Larson & Roughley

E714B002-6CE3-5EAE-A0F4-9E91B486C926

#### Notes.

This species is reported for the first time in Nova Scotia from eleven specimens collected in Cape Breton County, Inverness County and Victoria County (samples V12, V26, V27, C53, C59, I88, I90).

#### Habitat.

In Newfoundland and Prince Edward Island, this species has been collected from among flooded grasses and emergent *Carex* along the margins of beaver ponds and roadside ponds, which is similar to the habitats where these beetles were collected in Cape Breton Island which include also eutrophic creeks.

#### Distribution in the Maritime Ecozone.

This large, distinctive *Hydroporus* species has generally been confused with *Hydroporus rectus* Fall. In the Maritime ecozone, *H. gossei* is also reported from the neighboring province New Brunswick and Prince Edward Island (Larson et al. 2000; Bousquet et al. 2013; Alarie 2016).

### Hydroporus nigellus

Taxon classificationAnimaliaColeopteraDytiscidae

Mannerheim

EE26D99C-2111-5607-8514-7DCA644B6C7E

#### Notes.

This species is reported from only two specimens collected in Cape Breton County and Victoria County (samples C21, V28).

#### Habitat.

These beetles are common in small pools with dense emergent vegetation. The two specimens collected in Cape Breton Island were from a sphagnum bog and a eutrophic pool besides a river.

#### Distribution in the Maritime Ecozone.

Prior to this study, this species had only been reported from the neighboring province New Brunswick (Larson et al. 2000; Bousquet et al. 2013). The North American range of this Holarctic species includes most of the boreal zone and extends north to the southern arctic (Larson et al. 2000).

### Hydroporus puberulus

Taxon classificationAnimaliaColeopteraDytiscidae

LeConte

83D6593D-5114-5637-9DC9-2F4D6803F86C

#### Notes.

*Hydroporus puberulus* is reported from nine specimens form two localities in Cape Breton County and Inverness County (samples C53, I71).

#### Habitat.

These beetles were generally collected from small pools where the water is cool, such as small pools in bogs or habitats where the water is densely shaded by *Carex* (Larson et al. 2000), which is similar to the habitats where these beetles were collected in Cape Breton Island.

#### Distribution in the Maritime Ecozone.

In North America this Holarctic species occurs in the boreal zone from western Newfoundland to Alaska (Larson et al. 2000). Prior to this study, this species had only been reported from the neighboring province New Brunswick in the Maritime Ecozone (Larson et al. 2000; Bousquet et al. 2013).

### Ilybius picipes

Taxon classificationAnimaliaColeopteraDytiscidae

(Kirby)

D8761173-439C-5BF6-A53F-8FF8572DB12B

#### Notes.

*Ilybius picipes* is closely similar to *I. angustior* (Gyllehal) from which it can be differentiated by the relative expansion of the protarsal claw (Larson et al. 2000). In Cape Breton Island this species is reported from six specimens from two localities in Victoria County and Inverness County (samples V26, I76).

#### Habitat.

These beetles are generally collected from peatland pools (Larson et al. 2000), which is similar to the habitats where these beetles were collected in Cape Breton Island.

#### Distribution in the Maritime Ecozone.

This species has a Holarctic distribution. In North America it is transcontinental in the boreal region (Larson et al. 2000). Its presence in Cape Breton Island represents the first mention in the Canadian Maritimes (Bousquet et al. 2013).

### Ilybius wasastjernae

Taxon classificationAnimaliaColeopteraDytiscidae

(C.R. Sahlberg)

6937479A-A336-5252-B028-370C659CA09F

#### Notes.

*Ilybius wasastjernae* is reported in Cape Breton Island from only one specimen collected in Victoria County (sample V27).

#### Habitat.

These beetles are generally collected from sphagnum pools, usually in, or adjacent to forest (Larson et al. 2000). In Cape Breton Island it was collected in an ephemeral woodland pool covered with *Scirpus*.

#### Distribution in the Maritime Ecozone.

This species has a Holarctic distribution more or less throughout the boreal zone. In North America it is transcontinental in the boreal region (Larson et al. 2000). Prior to this study, this species had only been reported from the neighboring province New Brunswick in the Maritime Ecozone (Larson et al. 2000; Bousquet et al. 2013).

## Discussion

A total of 85 Hydradephaga species was recovered from 94 samples during a survey conducted on Cape Breton Island, Canada, between 2006–2007. According to this study and literature (Majka and Kenner 2009) 87 species of Hydradephaga are currently known from Cape Breton Island (Table 3). There are records of 48 species from Cape Breton County, 68 from Inverness County, 56 from Richmond County and 50 from Victoria County. The significantly larger number of species from both Inverness and Richmond Counties is noteworthy knowing that this region includes some of the most undisturbed areas in Nova Scotia.

**Table 3.** Checklist of species of Hydradephaga recorded from Cape Breton Island, Nova Scotia, Canada, and their provincial and territorial distribution within northeastern North America (NA). Key: asterisk (*), Holarctic species; cross (†), species not collected in this survey but recorded in Majka (2009); C, Cape Breton County; I, Inverness County; R, Richmond County; V, Victoria County; species in **bold** correspond to strict eastern Canada elements; i.e., never recorded west of the province of Ontario.

Taxon	Counties				Distribution in northeastern North America
	C	I	R	V	
** GYRINIDAE **					
** Gyrininae **					
Enhydrini					
*Dineutus hornii* Roberts	1	1	1		CT, MA, ME, MI, NB, NH, NS, NY, ON, PE, QC, RI
*Dineutus nigrior* Roberts	1		1		CT, MA, ME, MI, NB, NH, NS, ON, PE, QC, RI
Gyrinini					
*Gyrinus affinis* Aubé	1	1	1		LB, MA, ME, NB, NF, NH, NS, NY, ON, PE, QC, RI, VT
*Gyrinis aquiris* LeConte	1	1	1		LB, MA, ME, MI, NB, NF, NS, NY, ON, PE, QC, RI
*Gyrinus cavatus* Atton†		1			LB, MA, ME, NF, NH, NS, NY, ON, QC, RI
*Gyrinus confinis* Fall			1		CT, LB, MA, ME, NB, NF, NH, NS, NY, ON, PE, QC, SM, VT
*Gyrinus fraternus* Couper			1		MA, ME, NB, NH, NS, NY, ON, PE, QC, VT
***Gyrinus gehringi* Chamberlain**	1	1	1	1	**NB, NS, NF, NH, ON, PE, QC**
*Gyrinus impressicollis* Kirby†				1	NS, ON, QC
*Gyrinus latilimbus* Fall	1	1	1	1	CT, LB, MA, ME, NB, NF, NH, NS, NY, ON, PE, QC, SE
***Gyrinus pugionis* Fall**	1	1		1	**MA, ME, MI, NB, NH, NS, NY, ON, PE, QC. VT**
*Gyrinus sayi* Aubé	1	1	1	1	CT, MI, LB, MA, ME, NB, NF, NH, NS, NY, ON, PE, QC, RI, SM
** HALIPLIDAE **					
*Haliplus canadensis* Wallis	1	1			MA, NB, NS, ON, PE, QC
*Haliplus connexus* Matheson		1			CT, MA, ME, NB, NH, NS, NY, ON, PE, QC, VT
*Haliplus cribarius* LeConte		1	1		CT, LB, MA, ME, MI, NB, NF, NH, NS, NY, ON, PE, QC, SM
*Haliplus fulvus* (Fabricius)*	1	1	1		ON, MA, ME, NB, NF, NH, NS, NY, ON, QC
*Haliplus immaculicollis* Harris	1	1	1	1	CT, LB, MA, ME, MI, NB, NF, NH, NS, NY, ON, QC, PE, RI, SM, VT
*Haliplus longulus* LeConte		1	1		MA, ME, NB, NH, NB, NS, NY, ON, PE, QC, RI
*Peltodytes edentulus* (LeConte)		1			MA, NB, NH, NS, ON, QC, PE, RI
** DYTISCIDAE **					
Agabinae					
Agabini					
*Agabus ambiguus* (Say)	1	1		1	LB, ME, MI, NB, NF, NH, NS, ON, PE, QC, RI, SM
*Agabus anthracinus* Mannerheim	1	1	1	1	LB, MA, ME, MI, NB, NF, NH, NS, NY, ON, PE, QC, SM, VT
***Agabus erythropterus* (Say)**	**1**	**1**	**1**	**1**	**CT, LB, MA, ME, NB, NF, NS, NY, ON, PE, QC, RI**
*Agabus leptapsis* (LeConte)		1		1	LB, ME, NB, NF, NS, NY, ON, QC, VT
*Agabus phaeopterus* (Kirby)				1	LB, MA, ME, MI, NB, NF, NH, NS, NY, ON, PE, QC
*Agabus semipunctatus* (Kirby)	1		1	1	CT, LB, MA, ME, MI, NB, NF, NH, NS, NY, ON, QC, RI
*Agabus subfuscatus* Sharp	1	1	1		CT, LB, MA, ME, NB, NF, NH, NS, ON, PE, QC, VT
*Ilybiosoma seriatum* (Say)	1	1	1	1	CT, LB, MA, ME, MI, NB, NF, NH, NS, ON, PE, QC, SM
*Ilybius angustior* (Gyllenhal)*		1	1		LB, MI, ME, NB, NF, NH, NS, ON, PE, QC, SM, VT
*Ilybius biguttulus* (Germar)	1	1	1	1	MA, ME, MI, NB, NF, NH, NS, NY, ON, PE, QC, RI, SM, VT
***Ilybius confusus* Aubé**		**1**			**CT, MA, ME, NB, NH, NS, NY, ON, RI. VT**
*Ilybius discedens* Sharp*	1	1	1	1	LB, ME, MI, NB, NF, NH, NS, ON, PE, QC, SM
*Ilybius erichsoni* G. & H.*	1			1	LB, MA, ME, MI, NB, NF, NH, NS, NY, ON, PE, QC
***Ilybius ignarus* (LeConte)**	**1**		**1**	**1**	**CT, MA, ME, NH, NS, NY, ON, QC, RI**
***Ilybius larsoni* (Fery & Nilsson)**	**1**	**1**	**1**	**1**	**NB, NS, ON, PE, QC**
*Ilybius picipes* (Kirby)*		1		1	LB, NF, NS, ON, QC
*Ilybius pleuriticus* (Leconte)	1	1			CT, LB, MA, ME, MI, NB, NF, NS, ON, PE, QC, RI, SM, VT
*Ilybius wasastjernae* (C. R. Sahlberg)*				1	ME, LB, NB, NF, NS, ON, QC
***Platambus obtusatus* (Say)**	**1**	**1**	**1**	**1**	**CT, MA, ME, NB, NH, NS, NY, ON, QC, VT**
Colymbetinae					
Colymbetini					
*Colymbetes paykulli* Erichson*	1			1	LB, ME, NB, NF, NS, ON, PE, QC
*Colymbetes sculptilis* Harris		1			CT, LB, MI, NB, NF, NH, NS, NY, ON, PE, QC, RI
*Meridiorhantus sinuatus* (LeConte)			1	1	LB, MA, ME, NB, NF, NH, NS, NY, ON, PE, QC
*Rhantus binotatus* (Harris)		1	1	1	CT, LB, ME, MI, NB, NF, NH, NS, ON, PE, QC, RI, SM
*Rhantus suturellus* (Harris)*			1	1	CT, LB, MA, ME, MI, NB, NF, NH, NS, NY, ON, PE, QC, SM
*Rhantus wallisi* Hatch	1	1	1		LB, MA, MI, NB, NF, NH, NS, ON, PE, QC, SM
Copelatinae					
Copelatini					
***Copelatus glyphicus* (Say)**	**1**		**1**	**1**	**CT, ME, NF, NH, NB, NS, ON, PE, QC, RI**
Coptotominae					
Coptotomini					
***Coptotomus longulus* LeConte**	**1**		**1**		**MA, ME, MI, NB, NH, NS, NY, ON, QC, NB, PE, RI**
Dytiscinae					
Aciliini					
*Acilius mediatus* (Say)		1		1	CT, MA, NB, NH, NS, ON, PE, QC, RI
*Acilius semisulcatus* Aubé		1	1	1	CT, LB, MA, ME, MI, NB, NF, NH, NS, ON, PE, QC, RI, SM
Dytiscini					
*Dytiscus fasciventris* Say		1		1	CT, LB, ME, NB, NH, NS, ON, PE, QC, RI
*Dytiscus verticalis* Say		1	1		CT, MA, ME, NB, NH, NS, NY, ON, PE, QC, RI
Hydaticini					
*Hydaticus aruspex* Clark*	1	1	1	1	CT, LB, MA, ME, NB, NF, NH, NS, ON, PE, QC
Hydroporinae					
Bidessini					
***Liodessus affinis* (Say)**	**1**	**1**	**1**	**1**	**CT, ME, NB, NF, NH, NS, ON, PE, QC, RI**
Hydroporini					
***Heterosternuta allegheniana* (Matta & Wolfe)**		1	1		NB, **NS**, QC
***Heterosternuta pulchra* (LeConte)**		**1**	**1**	**1**	**CT, ME, LB, NB, NF, NS, ON, QC, SM**
***Heterosternuta wickhami* (Zaitzev)**		**1**		**1**	**NS, ON, QC**
*Hydrocolus paugus* (Fall)	1	1	1	1	LB, MA, ME, MI, NB, NF, NH, NS, NY, ON, PE, QC, SM
*Hydrocolus stagnalis* (G. & H.)	1	1	1	1	CT, MA, ME, NB, NH, NS, NY, ON, PE, QC
*Hydroporus appalachius* Sherman		1			LB, MA, ME, NH, NS, ON, QC
*Hydroporus badiellus* Fall		1	1	1	LB, ME, MI, NB, NF, NH, NS, ON, QC
*Hydroporus dentellus* Fall		1	1	1	LB, MA, ME, MI, NB, NH, NS, NY, ON, PE, QC
***Hydroporus gossei* Larson & Roughley**	**1**	**1**		**1**	**ME, NB, NF, NS, NY, ON, PE, QC**
*Hydroporus nigellus* Mannerheim*	1			1	LB, NB, NS, ON, QC
***Hydroporus niger* Say**	**1**	**1**			**CT, MA, MI, NB, NF, NH, NS, NY, ON, PE, QC, RI**
*Hydroporus notabilis* LeConte*	1	1	1	1	LB, MA, ME, MI, NB, NF, NH, NS, ON, PE, QC, SM
*Hydroporus obscurus* Sturm*		1			LB, NB, NF, NS, ON, PE, QC, SM
*Hydroporus puberulus* LeConte*	1	1			LB, ME, NB, NF, NS, ON, QC
*Hydroporus rufinasus* Mannerheim			1		ME, NB, NS, ON, QC
*Hydroporus signatus* Mannerheim	1		1	1	CT, LB, MA, ME, MI, NB, NF, NH, NS, NY, ON, PE, QC, RI, SM
*Hydroporus striola* (Gyllenhal)*	1	1	1	1	LB, ME, MI, NB, NF, NH, NS, ON, PE, QC, RI
*Hydroporus tenebrosus* LeConte	1	1			MA, ME, MI, NB, NH, NS, NF, ON, PE, QC,
*Hydroporus tristis* (Paykull)*	1	1		1	LB, ME, MI, NB, NF, NH, NS, NY, ON, PE, QC, RI, SM
*Nebrioporus rotundatus* (LeConte)	1	1	1		LB, MA, ME, NB, NF, NS, ON, PE, QC, RI
***Neoporus carolinus* (Fall)**	**1**	**1**	**1**	**1**	**LB, MA, ME, NB, NF, NH, NS, NY, ON, PE, QC, SM**
***Neoporus clypealis* (Sharp)**		**1**	**1**		**ME, NB, NH, NS, NY, ON, PE, QC**
*Neoporus dimidiatus* (G. & H.)		1		1	CT, LB, MA, ME, NB, NF, NH, NS, ON, PE, QC, RI
***Neoporus spurius* (LeConte)**		**1**		**1**	**NB, NS, ON, QC**
***Neoporus sulcipennis* (Fall)**		**1**	**1**		**NB, NH, NS, NY, ON, PE, QC**,
*Neoporus undulatus* (Say)	1	1	1	1	CT, LB, MA, ME, MI, NB, NF, NS, NY, ON, PE, QC, RI, SM
*Oreodytess. scitulus* (LeConte)		1			ME, LB, NB, NF, NH, NS, NY, ON, QC, SM
Hygrotini					
*Clemnius laccophilinus* (LeConte)		1			ME, NB, NH, NS, ON, PE, QC
*Hygrotus impressopunctatus* (Schaller)*			1		LB, ME, MI, NB, NF, NH, NS, ON, PE, QC
*Hygrotus picatus* (Kirby)	1	1	1	1	LB, MI, NB, NF, NH, NS, NY, ON, PE, QC, RI
*Hygrotus sayi* Balfour-Browne	1	1	1	1	LB, MA, ME, MI, NB, NF, NH, NS, NY, ON, PE, QC, RI, SM, VT
*Hygrotus turbidus* (LeConte)		1	1		MA, MI, NB, NH, NS, NY, ON, PE, QC, RI
Hyphydrini					
*Desmopachria convexa* (Aubé)	1	1	1		ME, MI, NB, NS, ON, PE, QC, RI
Laccornini					
***Laccornis latens* (Fall)**			**1**		**MA, NB, NH, NS, NY, ON, PE, QC**,
Laccophilinae					
Laccophilini					
*Laccophilus m. maculosus* Say	1	1	1	1	CT, MA, ME, MI, NB, NH, NS, ON, PE, QC, RI
Total	48	68	56	50	

**Notes**: Regional Distribution information derived from Downie and Arnett (1996), Larson et al. (2000), Majka (2008), Alarie (2009, 2016), Majka et al. (2011), Elder and Abraham (2012), Bousquet et al. (2013), and Webster (2016). Key: CT, Connecticut; LB, Labrador; MA, Massachusetts; ME, Maine; MI, Magdalen Island Archipelago, NB, New Brunswick; NF, insular Newfoundland; NH, New Hampshire; NS, Nova Scotia; NY, New York; ON, Ontario; PE, Prince Edward Island; QC, Québec; RI, Rhode Island; SM, Saint-Pierre et Miquelon; VT, Vermont.

Included among the species surveyed were eight new Nova Scotia records consisting of the dytiscid species *Heterosternuta allegheniana* (Matta & Wolfe), *H. wickhami* (Zaitzev), *Hydroporus appalachius* Sherman, *H. gossei* Larson & Roughley, *H. nigellus* Mannerheim, *H. puberulus* LeConte, *Ilybius picipes* (Kirby), and *I. wasastjernae* (C.R. Sahlberg) (Table 2). Among these, *Heterosternuta allegheniana* and *H. wickhami* stand out as representing the easternmost reports of these species in Canada.

The Nearctic component of the fauna is made up of 71 species (81.6%), the Holarctic component of 16 species (17.4%). Most species are characteristic of both the Boreal and Atlantic Maritime Ecozones and have a transcontinental distribution except for *Agabus erythropterus* (Say), *Copelatus glyphicus* (Say), *Coptotomus longulus* LeConte, *Heterosternuta allegheniana*, *H. pulchra* (LeConte), *H. wickhami*, *Hydroporus gossei*, *H. niger* Say, *Ilybius confusus* Aubé, *Ilybius ignarus* (LeConte), *I. larsoni* (Fery & Nilsson), *Laccornis latens* (Fall), *Liodessus affinis* (Say), *Nebrioporus rotundatus* (LeConte), *Neoporus carolinus* (Fall), *Neoporus clypealis* (Sharp), *N. spurius* (LeConte), *N. sulcipennis* (Fall), and *Platambus obtusatus* (Say), which are generally recognized as species with eastern affinities (Larson et al. 2000; Bousquet et al. 2013) (Table 3).

The 87 Hydradephaga species known from Cape Breton Island represent approximately 74% of the fauna known for all Nova Scotia (Bousquet et al. 2013). As is typical of Hydradephaga, the Dytiscidae accounts for the largest share (78.2%) of the fauna, while Gyrinidae (13.8%) and Haliplidae (8.1%) are represented in lesser proportions. Forty-five species were observed at more than 5% of the sites (Table 2). The most common ones (RFO > 15 %) included the haliplid species *Haliplus immaculicollis* Harris and the dytiscid species *Agabus ambiguus* (Say), *A. anthracinus* Mannerheim, *Hydroporus notabilis* LeConte, *H. signatus* Mannerheim, *H. striola* (Gyllenhal), *H. tristis* (Paykull), *Ilybiosoma seriatum* (Say), *Ilybius biguttulus* (Germar), *Laccophilus m. maculosus* Say, and *Neoporus carolinus* (Fall) (Table 2). Considering the relatively large number of species recorded in such a short time, confirm that this region is very important for wetland beetle biodiversity, and its continued conservation.

This research considerably extends the list of reported species in Richmond and Inverness counties both of which were considered the least well-known counties in all Nova Scotia in terms of its beetle fauna prior to this study. Many species recorded in this region are interesting in zoogeographic terms as representing new records for Nova Scotia (see above). The extraordinary species richness of rheophilic species (e.g., *Agabus leptapsis* (LeConte), *A. erythropterus* (Say), *Heterosternuta allegheniana*, *H. pulchra*, *H. wickhami*, *Hydroporus appalachius*, *Neoporus clypealis* (Sharp), *Neoporus dimidiatus* (G. & H.), *N. spurius*, *N. sulcipennis* (Fall), and *Oreodytess. scitulus* (LeConte)) are worth emphasizing in that they illustrate the high abundance of lotic habitats in this portion of Cape Breton Island. In general, coexisting species may be more closely related than expected by chance if environmental features of a given habitat select for certain traits that are shared by closely related species (Vamosi and Vamosi 2007), which may explain particularly the highest diversity of *Neoporus* Guignot and *Heterosternuta* Strand in that region.

The 87 species of Hydradephaga reported in this study represent an important richness, proportionally comparable to the number of species found in Prince Edward Island with similar environmental conditions (Alarie 2016). It is worth mentioning, however, that several species (*Agabus leptapsis*, *Dineutus hornii* Roberts, *Dytiscus fasciventris* Say, *Gyrinus cavatus* Atton, *G. fraternus* Couper, *G. gehringi* Chamberlain, *G. impressicollis* Kirby, *Haliplus fulvus* (Fabricius), *Heterosternuta allegheniana*, *H. pulchra*, *H. wickhami*, *Hydroporus appalachius*, *H. badiellus* Fall, *H. nigellus*, *H. puberulus*, *H. rufinasus* Mannerheim, *Ilybius ignarus*, *I. picipes*, *I. wasastjernae*, *Neoporus spurius*, *Oreodytess. scitulus*, and *Platambus obtusatus*) collected in Cape Breton Island have yet to be discovered in Prince Edward Island. The absence in Cape Breton Island of 12 species listed in the latter province (*Acilius sylvanus* Hilsenhoff, *Agabus punctulatus* Aubé, *Boreonectes griseostriatus* (DeGeer), *Dytiscus dauricus* Gebler, *D. harrisii* Kirby, *Graphoderus liberus* (Say), *G. perplexus* Sharp, *Gyrinus bifarius* Fall, *G. lecontei* (Hope), *Hygrotus compar* Fall, *Peltodytes tortulosus* Roberts, and *Sanfilippodytes planiusculus* (Fall)) is worth mentioning as it suggests that the number of Hydradephaga species on Cape Breton island may be even greater than suggested by this study.

## Conclusions

Our study adds considerably to the previous knowledge about Hydradephaga in the Canadian Maritimes, with eight new records for the province of Nova Scotia. The composition of the Cape Breton fauna reflects that of the Maritime Provinces as a whole. Whereas all the species found on Cape Breton Island have also been recorded in New Brunswick (Webster 2016) (except *Gyrinus cavatus* Atton, *Heterosternuta wickhami*, *Hydroporus appalachius*, and *Ilybius picipes*) the presence on Prince Edward Island (Alarie 2016) of 12 species not reported on Cape Breton Island suggests that additional species could potentially still be found.

## Supplementary Material

XML Treatment for Heterosternuta allegheniana

XML Treatment for Heterosternuta wickhami

XML Treatment for Hydroporus appalachius

XML Treatment for Hydroporus gossei

XML Treatment for Hydroporus nigellus

XML Treatment for Hydroporus puberulus

XML Treatment for Ilybius picipes

XML Treatment for Ilybius wasastjernae
